# Hole Dynamics in Photoexcited Hematite Studied with Femtosecond Oxygen K-edge X-ray Absorption Spectroscopy

**DOI:** 10.1021/acs.jpclett.2c00295

**Published:** 2022-05-05

**Authors:** Yohei Uemura, Ahmed S. M. Ismail, Sang Han Park, Soonnam Kwon, Minseok Kim, Hebatalla Elnaggar, Federica Frati, Hiroki Wadati, Yasuyuki Hirata, Yujun Zhang, Kohei Yamagami, Susumu Yamamoto, Iwao Matsuda, Ufuk Halisdemir, Gertjan Koster, Christopher Milne, Markus Ammann, Bert M. Weckhuysen, Frank M. F. de Groot

**Affiliations:** † Inorganic Chemistry and Catalysis, Debye Institute for Nanomaterials Science, Utrecht University, Universiteitslaan 99, Utrecht, 3584 CG, The Netherlands; ‡ Laboratory of Environmental Chemistry, Energy and Environment Research Division, Paul Scherrer Institut, Villigen, 5232, Switzerland; § European XFEL, Holzkoppel 4, Schenefeld, 22869, Germany; ∥ PAL-XFEL, Pohang Accelerator Laboratory, 77 Cheongam-Ro, Nam-Gu, Pohang, Gyeongbuk 37673, South Korea; ⊥ Institute for Solid State Physics, University of Tokyo, Kashiwa, Chiba 277-8581, Japan; # Graduate School of Material Science, University of Hyogo, Kamigori, Hyogo 678-1297, Japan; g Faculty of Science and Technology and MESA + Institute for Nanotechnology, University of Twente, P.O. Box 2171, Enschede, 7500 AE, The Netherlands; h SwissFEL, Paul Scherrer Institut, Villigen, 5232, Switzerland

## Abstract

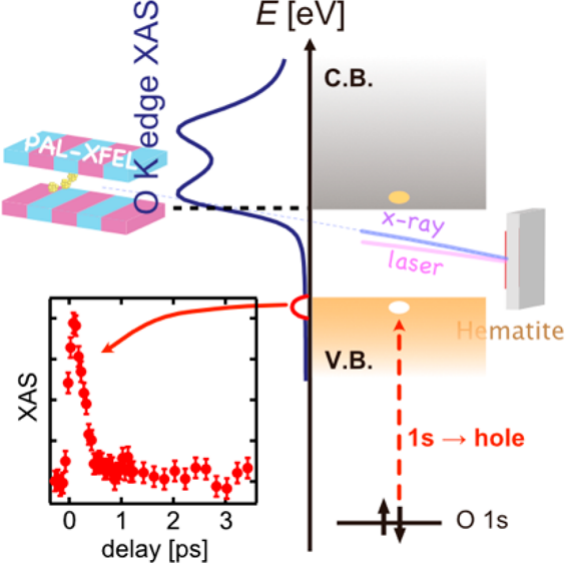

Hematite (α-Fe_2_O_3_) is a photoelectrode for the water splitting process because of its relatively narrow bandgap and abundance in the earth’s crust. In this study, the photoexcited state of a hematite thin film was investigated with femtosecond oxygen K-edge X-ray absorption spectroscopy (XAS) at the PAL-XFEL in order to follow the dynamics of its photoexcited states. The 200 fs decay time of the hole state in the valence band was observed via its corresponding XAS feature.

Photochemical water splitting reaction is a key process to generate hydrogen from water without any harmful products. Hematite (α-Fe_2_O_3_) is one of the promising photoelectrode materials for the solar-assisted water splitting process and has been studied intensively for decades. This is because iron is abundant in the earth’s crust,^1^ and its optical band gap is in the optimal range of absorption of sunlight.^2,3^ α-Fe_2_O_3_ is also an important photocatalytically active material associated with atmospheric mineral dusts impacting the oxidation capacity of the atmosphere.^4^ However, it is known that the intrinsic electronic properties of α-Fe_2_O_3_ can hinder its catalytic efficiency, in particular for the water splitting reaction. For example, its short hole diffusion length (<4 nm) suppresses the charge separation via electron–hole recombination.^5,6^ An in-gap state^7^ or a shift of band edge^8^ seems to be crucial for such low hole diffusion length. To understand its electronic properties under photoirradiation, theoretical calculations and ultrafast spectroscopic methods have been employed.^3,9−12^ It is well-known how the X-ray absorption spectroscopy (XAS) technique can address the element specific electronic structure of materials under investigation, and the recent advances of time-resolved XAS made possible the observation of excited states by photoabsorption in different systems.^10,13−20^ In addition, XAS can be obtained under in situ/operando conditions.^21,22^ By combining theoretical calculations with XAS experimental data a detailed picture of the local symmetry structure and the electronic states can be obtained.^13,23,24^ There have been pioneering works on investigating excited states of metal oxides by optical photoabsorption with the pump–probe XAS methodology.^10,13−20^ Furthermore, the birth of X-ray free electron lasers (XFEL) and the developments of X-ray sources by high-harmonic generation (HHG) have extended the scope the pump–probe XAS studies down to femtoseconds.^14,16,25−40^ Regarding the photoexcitation of α-Fe_2_O_3_, the first femtosecond extreme-ultraviolet (XUV) spectroscopic studies on α-Fe_2_O_3_ thin films were conducted by Vura-Weiss et al.^41^ Furthermore, it was suggested by theoretical and experimental XUV studies that localized carriers (small polarons), when being formed, behave as recombination centers, which can contribute to limiting the diffusion length of holes.^9,42−45^ We recently demonstrated that different processes occur in the early stage photoexcitation of α-Fe_2_O_3_ by measuring Fe L_3_ XAS and 2p3d resonant inelastic scattering (RIXS) in the Pohang Accelerator Laboratory XFEL (PAL-XFEL).^26^ Since the conduction band of α-Fe_2_O_3_ consists of Fe 3d orbitals mainly, while the valence band comprises O 2p orbitals, Fe L_3_ XAS and 2p3d RIXS can probe the conduction band after photoexcitation directly. The carrier relaxation and the charge recombination processes were observed from the viewpoint of the electronic state of the Fe 3d orbitals. The changes observed by Fe L_3_ XAS and 2p3d RIXS originate from the behavior of excited electrons in the conduction band.

Although previous transient XAS studies on the dynamics of α-Fe_2_O_3_ used X-ray absorption at Fe, in principle, it should be possible to observe transient features of oxygen XAS in the same time domains (<10 ps) because of its element selectivity. Since the valence band of α-Fe_2_O_3_ is mainly composed of oxygen 2p orbitals, oxygen K-edge XAS probes the holes in the valence band providing an effective avenue to investigate the dynamics of α-Fe_2_O_3_ after photoexcitation. Although there has been few reports on femtosecond XAS studies for lighter elements,^46−49^ to the best of our knowledge, oxygen K-edge XAS in condensed phases has not been yet investigated in femtoseconds due to the limitation of available X-ray sources and their properties such as X-ray energy, photon flux, and temporal resolution. Herein, we report the ultrafast dynamics of α-Fe_2_O_3_ as observed by oxygen K-edge XAS for the first time using the state-of-the-art PAL-XFEL.

Oxygen K-edge transient XAS (ΔXAS) spectra of α-Fe_2_O_3_ are shown in Figure 1A. An illustration of the experimental setup at the soft X-ray scattering and spectroscopy (SSS) beamline at PAL-XFEL is displayed in Figure 1D; a more detailed description of the setup is given in the experimental details section of the Supporting Information. To observe the photoexcited states of α-Fe_2_O_3_, the 50 nm thin film was excited by a femtosecond laser (wavelength, 400 nm; pulse duration, ∼70 fs). We note that hereafter, *“photoexcited”* state(s), electrons, and holes indicate the excited state, excited electrons, and excited holes, respectively, to avoid confusion. The repatition rate of XFEL was 30 Hz, and the laser was operated at 30 Hz and precisely synchronized with the XFEL. To observe the progress of the photoexcited state, the timing between the X-ray pulse and the laser pulse was changed using an optical delay stage. Each X-ray pulse from the XFEL hit the sample before (i.e., negative delay time) or after (i.e., positive delay time) the sample was excited by a laser pulse. ΔXAS at each delay time (Δ*t*) was calculated as ΔXAS = XAS(Δ*t*) – XAS(−5 ps). Since it required more than 1 h to obtain an XAS spectrum with a good signal-to-noise ratio, the long-term intensity fluctuations can affect the measurements slightly. To minimize this effect, XAS for a desired delay time and the reference XAS (Δ*t* = −5 ps) were measured in sequence, which successfully eliminates artifacts in the transient ΔXAS.^26^

**Figure 1:**
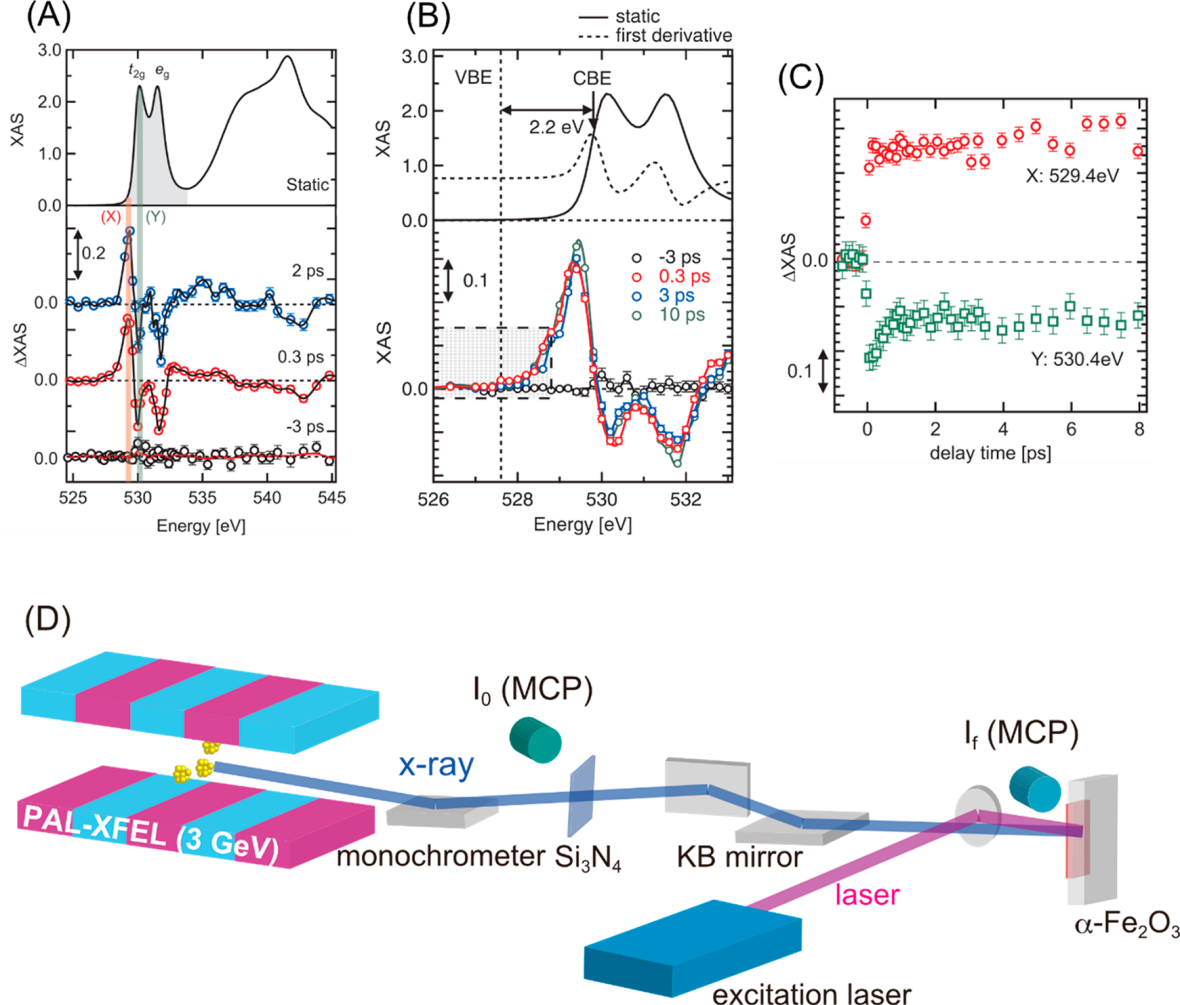
(A) Static oxygen K-edge XAS spectrum of α-Fe_2_O_3_ measured in SPring-8 (top) and oxygen K-edge ΔXAS of α-Fe_2_O_3_. Each ΔXAS is the difference between XAS at a delay time of *t* ps (*t* = −3, 0.3, or 2 ps) and that at a delay time of −5 ps. (B) O K-edge ΔXAS of α-Fe_2_O_3_ in the pre-edge region (526–534 eV). (C) Kinetic traces of difference XAS at 529.4 eV (X) and 530.2 eV (Y) shown in panel A. The conduction band edge (CBE) is at 529.8 eV while the valence band edge (VBE) is at 527.6 eV. (D) Illustration of the pump–probe XAS experimental setup in the SSS beamline, PAL-XFEL.

After photoexcitation, there were some distinctive changes observed (ΔXAS for 0.3 and 2 ps), in particular between 528 and 533 eV. Compared to the spectral features of the first derivative of the XAS of the ground state, the spectral features of the differential XAS are close to those of the first derivative (Figure S4), which implies that the main differences are attributed to the spectral shift of the O K-edge XAS. The sharp features of the XAS of the ground state (shown as shaded area in the top panel of Figure 1A) originate from electronic transitions from oxygen 1s to 2p orbitals that are hybridized with Fe 3d orbitals. The X-ray absorption features observed between 535 and 545 eV originate from oxygen 2p orbitals which are hybridized with Fe 4s/4p orbitals.^23^ In other words, the X-ray absorption between 528 and 533 eV reflects the oxygen p character of the iron 3d band of α-Fe_2_O_3_, and the absorption intensity between 528 and 533 eV is proportional to the oxygen p-projected density of the unoccupied states.^50^ To determine the dynamics of the photoexcited α-Fe_2_O_3_, the kinetic traces of XAS at the energies of 529.4 eV (denoted as X) and 530.4 eV (denoted as Y) were measured (see Figure 1C). Once the sample was excited by the optical laser, the XAS intensity at 529.4 eV increased while the XAS intensity at 530.4 eV decreased as shown in Figure 1C. These changes correspond to the initial photoexcitation to cause the spectral shift. At longer delay time (>0.2 ps), the XAS intensity at 530.4 eV shows a different trend from the XAS intensity at 529.4 eV. While the XAS intensity at 529.4 eV did not change, that is, no kinetic process was found, the XAS intensity at 530.4 eV decreased by a delay time of 1 ps. The decay process at 530.4 eV was fitted by a single exponential function *f*(*t*) = *A* + *B* exp(−*t*/τ) (A and B are arbitrary constants), and the time constant τ was estimated as 0.27 ± 0.06 ps.

To observe the behavior of electron holes after photoexcitation, we focused on the pre-edge area below 528.5 eV, that is, below the threshold energy of oxygen K-edge.^51^Figure 2A displays the shaded area of the bottom panel of Figure 1B (526.0–528.8 eV), where the valence band edge (VBE) is expected to be observed. Comparing the transient XAS at a delay time of 0.3 ps with the transient XAS at other delay times, a small increase of XAS was observed around the VBE area, which is a red-shaded area in Figure 2A. Since the XAS intensity is proportional to the density of unoccupied orbitals, the appearance of this peak implies that oxygen 1s electrons will be excited to the unoccupied O 2p orbitals below the energy threshold; that is, the direct electron transition to electron holes in the valence band was observed.

**Figure 2:**
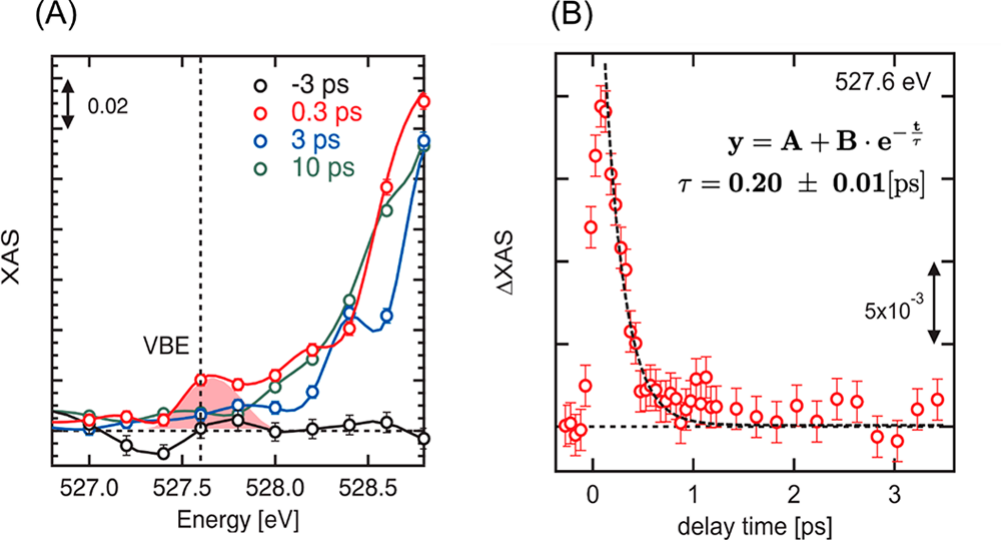
(A) Zoom of the pre-edge region shown as a dashed rectangle in Figure 1B. (B) Kinetic trace of XAS at 527.6 eV shown in Figure 1D. The dashed line is a single exponential function used for fitting.

The kinetic trace of XAS at 527.6 eV was also observed (Figure 2B). After the photoexcitation, the XAS intensity rose up immediately and it decreased within 1 ps. The decay process was fitted as a single exponential function, and the time constant was estimated as 0.20 ± 0.01 ps, which is a similar value estimated from the kinetic trace at 530.4 eV (Figure 1C). This result implies that the observed holes in the valence band disappeared within 1 ps by recombination or relaxation processes (we discuss this later in section II).

Hereafter, we would like to discuss the following topics: (I) Transient XAS features above the absorption edge, (II) transient signals below the absorption edge (at 572.6 eV), and (III) the fast kinetic process observed at 529.8 eV.

## I Transient XAS Features above the Absorption Edge

The transient XAS feature shown in Figure 1A is supposed to originate from the formation of Fe(II), which causes the spectral shift in the direction of lower energy. We note that while the L_3_ edge XAS of a metal ion shifts in higher energy by approximately 1.5 eV when its valence state changes from 2+ to 3+,^52^ the oxygen K-edge XAS of transition metal oxides does not have a general trend. For instance, the oxidation of Fe oxides hardly shifts the oxygen K-edge position in bulk oxides while the oxygen K-edge XAS of Cu and Ni oxides shifts to lower energy upon oxidation.^50,53,54^

After the optical excitation, we identify the following two events: (1) A change in the charge density of Fe atoms that shifts the iron 3d-band energy to lower energies and (2) a decrease of the unoccupied states of the Fe 3d orbitals.

1. A change of the charge density. An initial photoexcited state is created by the optical laser excitation, creating enhanced charge density in the (spin-down) iron 3d-band. The enhanced charge density shifts the iron 3d-band to lower energy, and the oxygen contribution to the iron 3d-band will also shift to lower energy. This effect results in the spectral shift mentioned above.
2. A decrease of the unoccupied state of Fe 3d orbitals. The X-ray absorption between 528 and 533 eV reflects the density of unoccupied Fe 3d orbitals; that is, the X-ray absorption is proportional to (the oxygen character of) the unoccupied Fe 3d orbitals. Since an Fe atom in α-Fe_2_O_3_ is surrounded by six oxygen atoms (nearly octahedral symmetry), its 3d orbitals are split into two different orbitals groups. The orbitals at the lower energy are t_2g_ orbitals, whereas those at the higher energy are e_g_ orbitals. The X-ray absorption spectra between 528 and 533 eV shows two peak features, which are assigned to t_2g_ and e_g_ orbitals, respectively.^50^ The energy difference between the t_2g_ and e_g_ peak corresponds to the crystal field splitting of the 3d orbitals of Fe in α-Fe_2_O_3_. The splitting in Figure 1A was 1.4 eV which is in agreement with previous reports.^50,51,53^ Upon photoexcitation, electrons are excited to the conduction band of α-Fe_2_O_3_, which is mainly composed by Fe 3d orbitals. In other words, the Fe 3d orbitals are filled by photoexcited electrons so that the X-ray absorption intensity between 528 and 533 eV after photoexcitation decreases.

The photoexcited state undergoes a decay process (∼0.2 ps as seen in Figure 1C) and forms a longer lived metastable state. Also, the metastable state has increased charge density with respect to the static Fe^3+^ state. This metastable state could involve reorganization of atoms related to a polaronic state.^44^

## II Transient Signals below the Absorption Edge (at 572.6 eV)

Gilbert et al. determined the band edge position of α-Fe_2_O_3_ from its oxygen K-edge XAS. To determine the edge position of the conduction band, they used the first derivative of the oxygen K-edge XAS, i.e. they defined the conduction band edge (CBE) as the maximum of the first derivative. On the other hand, the VBE position was defined by the first derivative of X-ray emission spectra (XES) of α-Fe_2_O_3_. According to this definition, they estimated the band gap energy as 2.2 eV, which is in good agreement with a standard value of the band gap of α-Fe_2_O_3_. We position the VBE by ∼2.2 eV below the conduction band edge. In the top panel of Figure 1B, a static oxygen K-edge XAS and its first derivative are displayed. If we calculate the CBE as the maximum of the first derivative, we position it around 529.8 eV. The energy difference between the maximum of the first derivative, that is, the CBE position and the transient peak position shown in Figure 2A is ∼2.2 eV, which is in agreement with the band gap value of α-Fe_2_O_3_. We conclude that the transient peak shown in Figure 2A can be ascribed to the transition of O 1s electrons to O 2p hole states. A similar peak was observed in the oxygen K-edge XAS of a hematite thin film. Braun et al. measured the oxygen K-edge XAS of a photoelectrode consisting of a hematite thin film under photoelectrochemical water splitting reaction, and a small peak appeared 2.2 eV far from the energy threshold.^55^ They assigned the peak to an O 2p-type hole.

A fast process with a kinetic constant of 0.2–0.3 ps was reported in the literature.^12,26,41,42,56^ This fast process was supposed to be a carrier relaxation, band filling, or band shrinking process. From our results, a fast charge recombination process can be involved in this early stage of photoexcitation. The XAS of the hole state at 527.6 eV disappeared and the line width of the difference signals between 528 and 533 eV did not change. However, since the XAS shifts in lower energy at later delays (>1 ps), there are certain amounts of electrons and holes in the conduction band and the valence band, respectively. We assume that the remaining holes distribute broadly in energy and shift in higher energy. Since the absorption of 1s → 2p excitation can be small and the distribution of the hole states becomes broad, an obvious peak of the hole state was not observed at the longer delays. The absorption of the hole states may be overlapped with the peaks at higher energy, which is another reason why the hole state was not observed at longer delays. The recombination below 1 ps may be affected by the excess carrier density.

## III The Fast Kinetic Process Observed at 529.8 eV

A fast kinetic process with a similar time constant was observed in our previous study of the transient Fe L_3_ XAS of the photoexcited α-Fe_2_O_3_.^26^ This fast process was supposed to be a relaxation process of “hot” electrons and holes created by the initial photoexcitation.^11,41,56,57^ Since the wavelength of the excitation laser was 400 nm (3.1 eV), which is greater than the bandgap of α-Fe_2_O_3_, the electrons and holes have excess energy and they relax to the lower energy level releasing their excess energy. The increase of peak Y after 1 ps can be ascribed to a change in hybridization between Fe 3d orbitals and O 2p orbitals driven by the occupation change occurring in the conduction band.

Considering the increase of the peak intensity at 530.4 eV at a delay of 1 ps, the change of the peak intensity can be explained by the decrease of the excited electrons in the t_2g_ orbitals. Since the holes in α-Fe_2_O_3_ have a limited mobility, a certain number of the holes in the valence band can encounter the electrons and recombine with each other, resulting in a decrease in the population of electrons in the conduction band. Both interpretations seem to be possible for the decay process at 530.4 eV. (It should be noted that a certain amount of electrons and holes exist in the conduction band and the valence band, respectively.)

**Figure 3:**
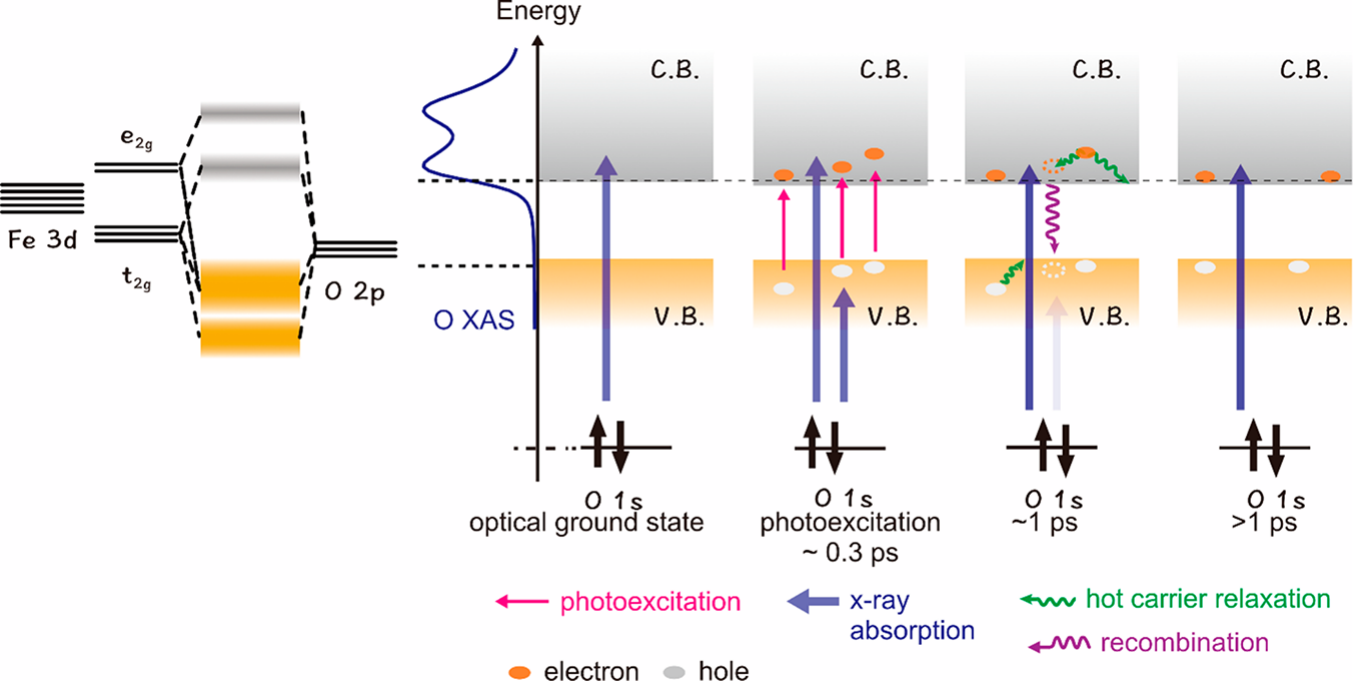
An illustration for the electronic state of the photoexcited α-Fe_2_O_3_ from the viewpoint of O K-edge XAS. The conduction band is denoted as C.B. and the valence band is as V.B.

In summary, the dynamics of the electronic state of the photoexcited α-Fe_2_O_3_ is illustrated in Figure 3. In the optical ground state, the oxygen K-edge XAS reflects the density of unoccupied Fe 3d orbitals. After the photoexcitation, electrons in the valence band are excited to the conduction band and holes are created in the valence band. The oxygen K-edge spectrum of the photoexcited α-Fe_2_O_3_ is shifted to lower energy because of the creation of Fe species with excess charge density and change of the hybridization between O 2p orbitals and Fe 3d orbitals. Hot carrier relaxation occurs and the kinetic constant for this process was estimated as ∼0.2 ps from the disappearance of the hole states in the valence band and the increase of the XAS intensity for t_2g_ orbitals. In this stage, the hole state disappears as electron–hole recombination, hole’s trapping into another energy level, and so on occur. It is supposed that trapped holes/electrons are more likely to be recombined with electrons/holes. The O 2p hole state observed by the transient oxygen K-edge XAS could be trapped hole states; that is, they are distributed in a narrow band in energy and localized in space. In the later delay times (>1 ps), free electrons and holes still exist.

Carnerio et al. observed small polaron formation by a delay time of 1 ps by femtosecond extreme-ultraviolet spectroscopy^44^ and we observed a relatively slow dynamics (*t* ∼ 6 ps),^26^ where the local structure of Fe should change. However, we did not find any signature for these dynamics. This implies that the transient oxygen K-edge XAS reflects the electronics state of Fe 3d orbitals more and the local structural change around the photoexcited Fe atoms does not have much influence on the oxygen K-edge XAS.

We successfully observed transient O K-edge XAS of photoexcited hematite and found several distinctive changes. The main differential spectral feature originates from the formation of Fe(II) species after photoexcitation, and observed dynamics were consistent with our previous study. At 527.6 eV, we found another feature which is associated with the XAS of 1s → 2p hole states and observed a fast decay process. The decay at 527.6 eV suggests the electron–hole recombination can happen below 1 ps. We suppose that the hole states we observed at 527.6 eV are localized in space and energy. At the early state of the photoexcitation, some of the holes can be recombined with electrons within 1 ps.

An advantageous point of pump–probe XAS is the determination of the energy level of carriers against the band edge positions (or absorption edge positions). Using pump–probe XAS can make it possible to figure out the dynamics of photocarriers according to their energy levels. To study more details of the dynamics of α-Fe_2_O_3_, resonant inelastic X-ray scattering (RIXS) will be helpful.^26,36^ Transient RIXS will potentially distinguish energy levels of the conduction band, midgap state, and valence band with an energy resolution of 100 meV (or less). Employing transient soft X-ray absorption spectroscopy can determine the electronic states of elements in metal oxides and reveal the dynamics of photoexcited materials.

## Experimental Details

The sample preparation, basic characterization of the sample, and the details of the pump probe experiments at PAL-XFEL are described in the Supporting Information.

## Supporting Information Available

Sample preparation, basic characterizations of the hematite thin film, the details of the pump–probe setup at PAL-XFEL, the data analysis of X-ray absorption and the evaluation of the temporal resolution at the beamline SSS, PAL-XFEL, and the comparison between transient XAS and the first derivative of the static O K-edge XAS (PDF)
