# Supplementary material for: Hole Dynamics in Photoexcited Hematite Studied with Femtosecond Oxygen K-edge X-ray Absorption Spectroscopy
Source: J Phys Chem Lett. 2022 May 5;13(19):4207–14. doi: 10.1021/acs.jpclett.2c00295 (PMC9125685; doi:10.1021/acs.jpclett.2c00295)
Supplement: Supplementary file 1 — jz2c00295_si_001.pdf [file jz2c00295_si_001.pdf]

Supporting Information

Hole Dynamics in Photoexcited Hematite Sstudied with Femtosecond Oxygen K-edge X-ray Absorption Spectroscopy

Yohei Uemura,[a,b,c] Ahmed S. M. Ismail,[a] Sang Han Park,[d] Soonnam Kwon,[d] Minseok Kim,[d] Hebatalla Elnaggar,[a] Federica Frati,[a] Hiroki Wadati,[e,f] Yasuyuki Hirata,[e] Yujun Zhang,[e] Kohei Yamagami,[e] Susumu Yamamoto,[e] Iwao Matsuda,[e] Ufuk Halisdemir,[g] Gertjan Koster,[g] Christopher Milne[c,h], Markus Ammann,[b] Bert M. Weckhuysen[a] and Frank M. F. de Groot*[a]

a. Inorganic Chemistry and Catalysis, Debye Institute for Nanomaterials Science, Utrecht University, Universiteitslaan 99, 3584 CG Utrecht, The Netherlands
b. Laboratory of Environmental Chemistry, Energy and Environment Research Division, Paul Scherrer Institut, Villigen 5232, Switzerland
c. European XFEL, Schenefeld 22869, Germany
d. PAL-XFEL, Pohang Accelerator Laboratory, 77 Cheongam-Ro, Nam-Gu, Pohang, Gyeongbuk 37673, South Korea
e. Institute for Solid State Physics, University of Tokyo, Kashiwa, Chiba 277-8581, Japan
f. Graduate School of Material Science, University of Hyogo, Kamigori, Hyogo 678-1297, Japan.
g. Faculty of Science and Technology and MESA+ Institute for Nanotechnology, University of Twente, P.O. Box 2171, 7500 AE Enschede, the Netherlands
h. SwissFEL, Paul Scherrer Institut, 5232 Villigen, Switzerland

Hematite Thin Film Fabrication and Characterization

Sample preparation: The 50 nm α-Fe_2O_3 thin-film was prepared by Pulsed Laser Deposition (PDL) as described in ref ref 1: first, the indium tin oxide (ITO)/fused SiO_2 substrate was prepared by translating a KrF excimer laser (λ = 248 nm) on a rotating ITO target (the laser fluency: 2 J cm^{-2}, the repetition rate: 1 Hz). Then, α-Fe_2O_3 was deposited on the substrate at the same deposition conditions using an α-Fe_2O_3 target instead of ITO. During the deposition, the substrate temperature was kept at 700°C and oxygen gas was introduced into the preparation chamber and its pressure was 1.3 kPa. The formation of α-Fe_2O_3 was confirmed by X-ray Photoelectron spectroscopy (XPS) and the average surface roughness of the thin-film

was estimated as 4.7 nm using Atomic Force Microscopy. Those basic characterisations of the sample were shown in the supplemental materials of ref ref 1.

50nm hematite thin film sample was deposited on indium tin oxide (ITO)/fused silicon dioxide substrate by pulsed laser deposition. The thin film surface morphology was studied with

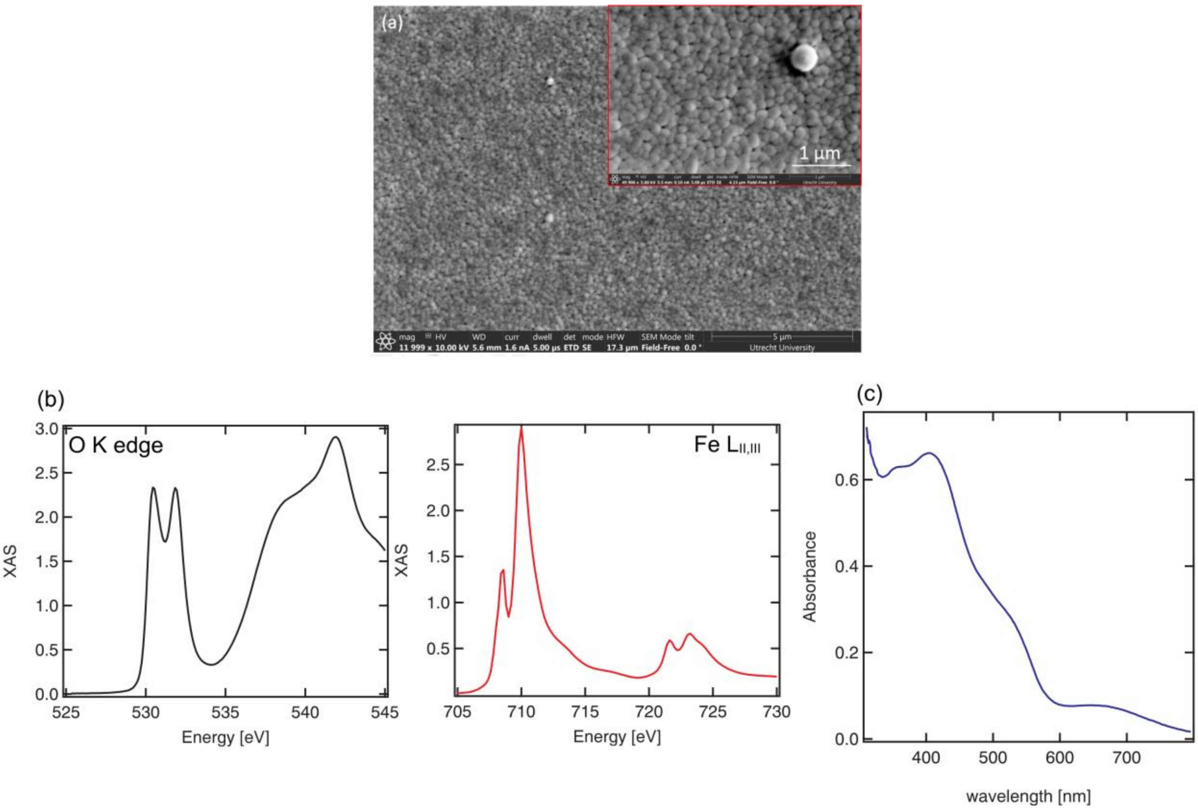

Figure S1: The basic characterizations of the prepared hematite sample: (a) SEM image (b) oxygen K edge and Fe $L_{II,III}$ edge XAS of the hematite thin film (c) a UV-Vis spectrum

scanning electron microscopy (SEM) (Fig. S1 (a)). Several different spots in the samples were investigated to ensure the surface homogeneity. Oxygen K-edge and iron $L_{II\text{-}III}$ edge X-ray absorption spectroscopy (XAS, Fig. S1 (b)) were measured in SPring-8 BL07LSU to confirm the chemical states of both elements. The spectral features of both measurements were consistent with those of a bulk sample. In addition, we measured X-ray photoelectron spectrum of the thin film sample, which suggested that the valence of the iron atoms in the sample should be 3+ (ref. ref 1). The electronic transitions and band gaps of the sample were estimated using a UV-VIS-NIR spectrometer (Fig. S1 (c)). Both transmittance and absorption spectra were recorded from 800 nm to 350 nm using a UV-vis-NIR Cary 500 (Varian) spectrophotometer.

The pump probe experiments at PAL-XFEL

All oxygen K edge XAS spectra were measured in the soft X-ray spectroscopy and scattering (SSS) beamline of PAL-XFEL. [1-4] In general, we employed the same experimental setup described in ref ref 1 except for the data acquisition part. The x-ray was scanned between 520 and 545 eV in order to measure the XAS spectra. The estimated energy resolution for this energy range was 0.5 eV. The X-ray was focused by a pair of Kirkpatrik-Baez (KB) mirrors to be less than 50 μm(H) × 50 μm(V) on the sample position. The incident x-ray intensity (I_0) for each x-ray shot was estimated by counting the electrons emitted from a Si_3N_4 thin-film by using a microchannel plate (MCP) detector while the fluorescence x-ray intensity (I_f) for each shot measured by using another MCP detector. The 2 channel digitizer (Adq7DC, Teledyne SP Devices, Linköping, Sweden), was synchronised with the trigger signal from the XFEL in order to gather only the signals from the X-ray pulses. The digitizer integrated the signals with an integration window of ~30 ns. The digitiser can accumulate the signals with a sampling rate of $5x10^9$ points/second (5 GSPS) and the vertical resolution is 14 bits. The digitizer reduced the readout noises to improve the signal-to-noise ratio compared to the previous experiments. [1-2] A Ti:Sapphire laser (λ: 800 nm, pulse duration: 35 fs) was employed to excite the sample. The

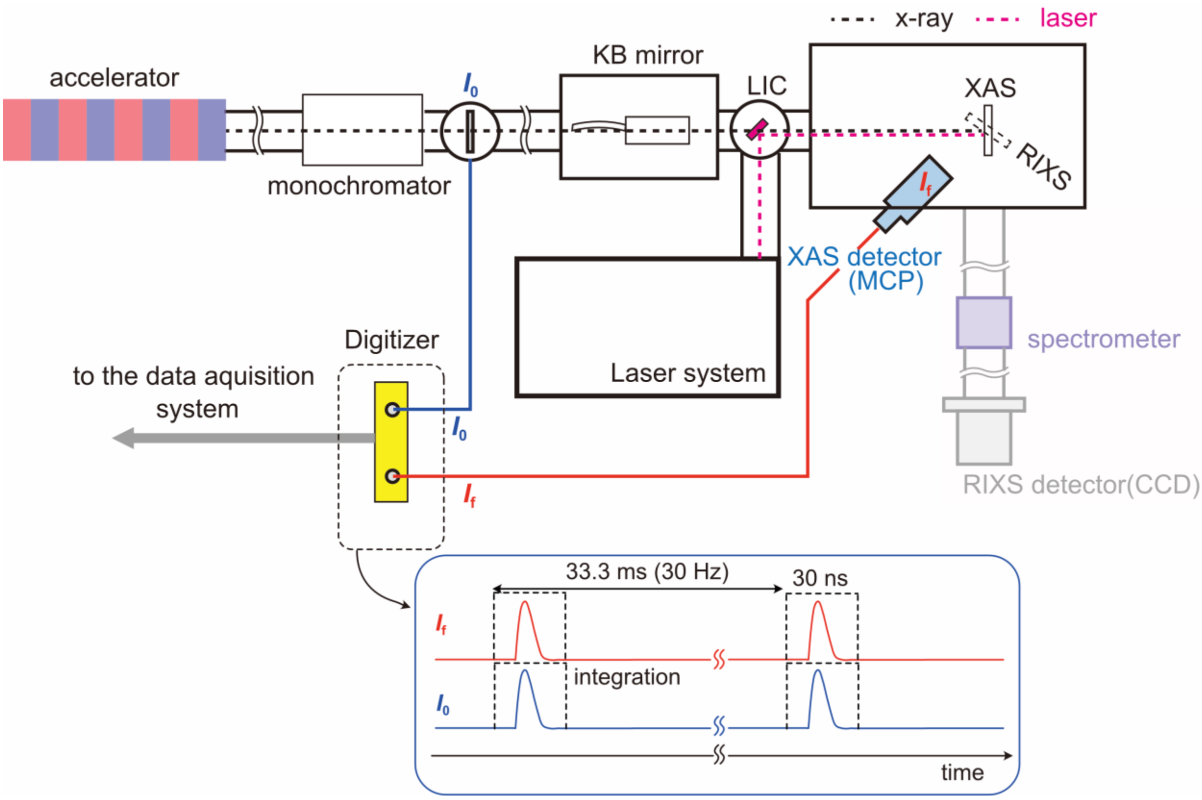

Figure S2: A detailed illustration of the setup and data acquisition scheme at the SSS beamline, PAL-XFEL

optical laser light was converted to 400 nm with a beta barium borate (BBO) crystal, transferred into the measurement chamber and focused on the sample position (the spot size was approximately 210 μm(H) × 210 μm(V) for its $1/e^2$ value). The pulse duration of laser is doubled to have 70 fs traveling BBO crystal. The angle between the X-ray and the laser was set to about 1° and the excitation laser fluence was set to ~8 mJ cm^{-1}. Static XAS data was measured at BL07LSU, SPring-8, Japan. The measurement was conducted using the setup described in ref. ref 5. The incident x-ray intensity was monitored by the drain current of the mirror and the XAS signals were corrected by measuring sample currents.

Analysis of X-ray absorption data in PAL-XFEL

Since the intensity of each x-ray pulse fluctuates a lot at PAL-XFEL because of its intrinsic property (Self-Amplified Spontaneous Emission (SASE)), the data acquisition system at the SSS beamline was designed to have a wide dynamic range and acquire each x-ray pulse intensity. However, some of x-ray pulses have too low or large intensity that are above or below the range of the data acquisition system. In order to measure static XAS with a good signal-to-noise ratio (S/N), eliminating x-ray pulses that are out of range of the system is a crucial step. The lower and upper thresholds for I_0 were set to 1% of the digitizer's maximum input valueand 99 % of the maximum value, respectively (the same thresholds were applied for I_f). In addition to the thresholds, the average (avg_{XAS}) and the standard deviation (s_{XAS}) of I_f/I_0, which corresponds to XAS for each x-ray pulse, was used for the analysis. If the absolute value of I_f/I_0 - avg_{XAS} for an x-ray pulse was less than 2*s_{XAS}, the x-ray pulse was included for the calculation of XAS. According to these thresholds, we accumulated typically more than 500 shots for each energy or delay point. The ratio of the transient signals to the statistical error was estimated as more than 10.

Temporal resolution at the SSS beamline

The temporal resolution was estimated by fitting the fast XAS changes at 527.8 eV and 529.4 eV shown in Fig 1 in the main text. Each rising slope was fitted by a convolution of a step function and a gaussian function as below.

$$f_{\mathsf{con}}(t) = F_{\mathsf{step}}(t) \otimes G(t)$$

$$F_{\mathsf{step}}(t) = \begin{cases} 0 & \text{for} \quad t < 0 \\ 1 & \text{for} \quad t \geq 0 \end{cases}$$

$$G(t) = \frac{1}{\sqrt{2\pi\sigma}} \exp\left(-\frac{t^2}{2\sigma^2}\right)$$

The temporal resolution (TR) was estimated from the value of σ: $TR \simeq 2.355\sigma$. The results of the fittings are shown in Fig. S3. The best fitting results were obtained when the value of σ was 63.7 [fs] which corresponds to TR = 150 fs. Considering our previous results [1-2], the estimated temporal resolution is reasonable. The pulse width of the x-ray at the SSS beamline is estimated as > 100 fs and the pulse width of the excitation laser is > 70 fs. Therefore, the minimum temporal resolution should be > 122 fs if there were no timing jitters between the x-ray and the laser. We obtained a good temporal resolution during our experiments owing to the good synchronization that yields very small jitters at the SSS beamline.

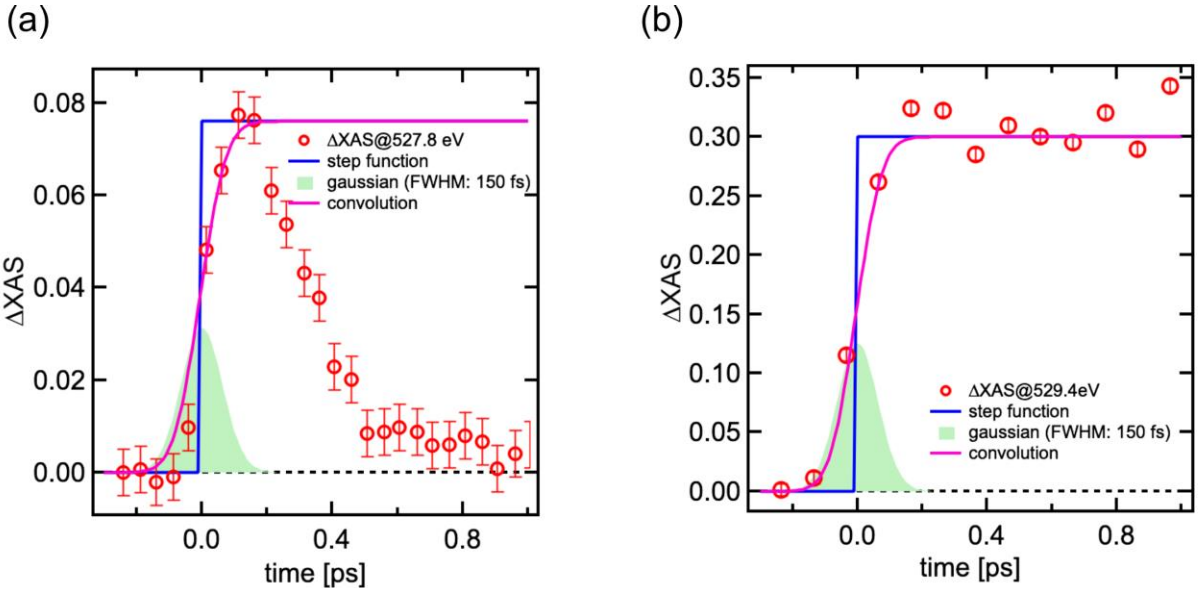

Figure S3: Fittings of the rising of XAS at 527.8 eV (a) and at 529.4 eV using a convolution of a step function and a gaussian function. The best fit results were obtained if the Full with at half maximum (FWHM) was 150 fs.

Comparison of the difference spectra

Figure S4 displays the comparison of the first derivative of the static XAS of α-Fe_2O_3 and the differential XAS (0.3 ps and 2 ps). The spectral features of the differential XAS are close to the first derivative of the static XAS spectrum. It is supposed that the differential XAS reflects the spectral shift of the ground state spectrum.

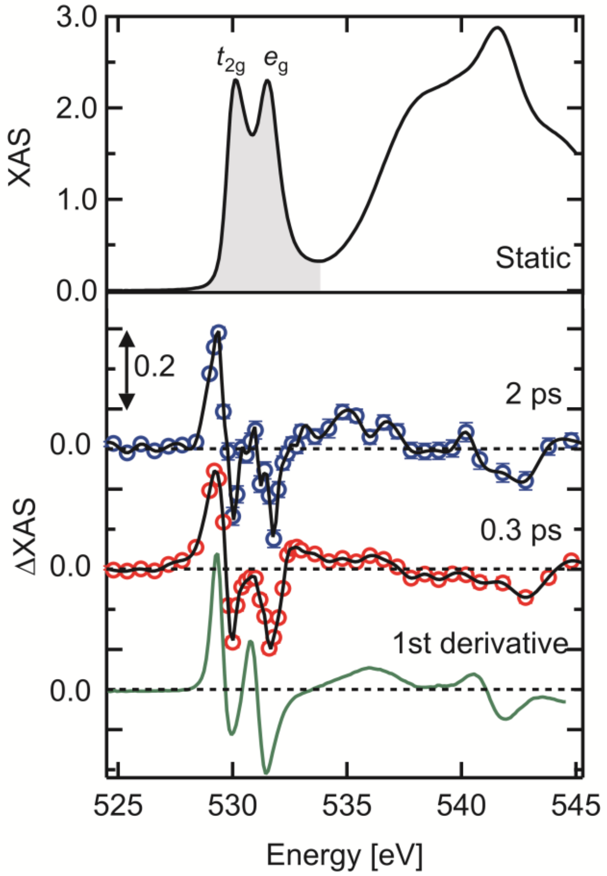

Figure S4: Comparison between the difference XAS (0.3 ps and 2 ps) and the first derivative of the static XAS

References

(1) Ismail, A. S. M.; Uemura, Y.; Park, S. H.; Kwon, S.; Kim, M.; Elnaggar, H.; Frati, F.; Niwa, Y.; Wadati, H.; Hirata, Y.; Zhang, Y.; Yamagami, K.; Yamamoto, S.; Matsuda, I.; Halisdemir, U.; Koster, G.; Weckhuysen, B. M.; de Groot, F. M. F. Direct observation of the electronic states of photoexcited hematite with ultrafast 2p3d X-ray absorption spectroscopy and resonant inelastic X-ray scattering. *Phys. Chem. Chem. Phys.* **2020,** *22*, 2685-2692, 10.1039/C9CP03374B.

(2) Uemura, Y.; Ismail, A. S. M.; Park, S. H.; Kwon, S.; Kim, M.; Niwa, Y.; Wadati, H.; Elnaggar, H.; Frati, F.; Haarman, T.; Höppel, N.; Huse, N.; Hirata, Y.; Zhang, Y.; Yamagami, K.; Yamamoto, S.; Matsuda, I.; Katayama, T.; Togashi, T.; Owada, S.; Yabashi, M.; Halisdemir, U.; Koster, G.; Yokoyama, T.; Weckhuysen, B. M.; de Groot, F. M. F. Femtosecond Charge Density Modulations in Photoexcited $CuWO_4$. *J. Phys. Chem. C* **2021,** *125*, 7329-7336, 10.1021/acs.jpcc.0c10525.

(3) Park, S. H.; Kim, M.; Min, C.-K.; Eom, I.; Nam, I.; Lee, H.-S.; Kang, H.-S.; Kim, H.-D.; Jang, H. Y.; Kim, S.; Hwang, S.-m.; Park, G.-S.; Park, J.; Koo, T.-Y.; Kwon, S. PAL-XFEL soft X-ray scientific instruments and X-ray optics: First commissioning results. *Rev. Sci. Instrum.* **2018,** *89*, 055105, 10.1063/1.5023557.

(4) Kang, H.-S.; Min, C.-K.; Heo, H.; Kim, C.; Yang, H.; Kim, G.; Nam, I.; Baek, S. Y.; Choi, H.-J.; Mun, G.; Park, B. R.; Suh, Y. J.; Shin, D. C.; Hu, J.; Hong, J.; Jung, S.; Kim, S.-H.; Kim, K.; Na, D.; Park, S. S.; Park, Y. J.; Han, J.-H.; Jung, Y. G.; Jeong, S. H.; Lee, H. G.; Lee, S.; Lee, S.; Lee, W.-W.; Oh, B.; Suh, H. S.; Parc, Y. W.; Park, S.-J.; Kim, M. H.; Jung, N.-S.; Kim, Y.-C.; Lee, M.-S.; Lee, B.-H.; Sung, C.-W.; Mok, I.-S.; Yang, J.-M.; Lee, C.-S.; Shin, H.; Kim, J. H.; Kim, Y.; Lee, J. H.; Park, S.-Y.; Kim, J.; Park, J.; Eom, I.; Rah, S.; Kim, S.; Nam, K. H.; Park, J.; Park, J.; Kim, S.; Kwon, S.; Park, S. H.; Kim, K. S.; Hyun, H.; Kim, S. N.; Kim, S.; Hwang, S.-m.; Kim, M. J.; Lim, C.-y.; Yu, C.-J.; Kim, B.-S.; Kang, T.-H.; Kim, K.-W.; Kim, S.-H.; Lee, H.-S.; Lee, H.-S.; Park, K.-H.; Koo, T.-Y.; Kim, D.-E.; Ko, I. S. Hard X-ray free-electron laser with femtosecond-scale timing jitter. *Nat. Photonics* **2017,** *11*, 708-713, 10.1038/s41566-017-0029-8.

(5) Takubo, K.; Yamamoto, K.; Hirata, Y.; Yokoyama, Y.; Kubota, Y.; Yamamoto, S.; Yamamoto, S.; Matsuda, I.; Shin, S.; Seki, T.; Takanashi, K.; Wadati, H. Capturing ultrafast magnetic dynamics by time-resolved soft x-ray magnetic circular dichroism. *Appl. Phys. Lett.* **2017,** *110*, 162401, 10.1063/1.4981769.
